# Sidedness determines clinical characteristics and survival outcomes in medullary adenocarcinoma of the colon

**DOI:** 10.1038/s41598-021-99848-y

**Published:** 2021-10-14

**Authors:** Andrew M. Blakely, Rebecca A. Nelson, Stanley A. Hamilton, Lily L. Lai

**Affiliations:** 1 Surgical Oncology Program, National Cancer Institute, Bethesda, MD USA; 2 Department of Computational and Quantitative Medicine, City of Hope National Medical Center, Duarte, CA USA; 3 Department of Pathology, City of Hope National Medical Center, Duarte, CA USA; 4 Department of Surgery, City of Hope National Medical Center, 1500 East Duarte Road, Duarte, CA 91010 USA

**Keywords:** Cancer, Cancer epidemiology

## Abstract

Colon medullary adenocarcinoma (MAC) is a rare histologic subtype. Clinical presentation and cancer outcomes of MAC, compared to colon adenocarcinoma (AC), remain incompletely described. Annual age-adjusted incidence rates were computed using Surveillance, Epidemiology, and End Results (2002–2017). A cohort analysis using the National Cancer Database (2010–2016) compared patient characteristics in an unmatched dataset and prognostic characteristics in a 1:1 matched subset. Reported annual age-adjusted incidence of MAC has significantly increased, with an average annual percent change (APC) increase of 23.8% (95% CI: 19.2–28.6); concurrent AC incidence declined (APC: − 2.8, 95% CI: − 3.1 to − 2.8). Analyses of 1018 MAC and 210,784 AC unmatched patients showed that MAC patients were more often older, female, and white, with higher disease stage, poorly-differentiated tumors, right-sided laterality, and lymphovascular invasion (all *p* < 0.0001). Among those with known microsatellite status, instability was more prevalent among MAC than AC patients (82% vs. 24%, *p* < 0.0001). Multivariate analyses of the matched dataset revealed that MAC histology was not independently associated with overall survival. However, when stratifying by laterality, left-sided MAC was associated with shorter survival when compared to right-sided MAC (HR 1.66, 95% CI 1.16–2.38) and right-sided AC (HR 1.54, 95% CI 1.12–2.12). The reported incidence of MAC is increasing, in contrast to the declining incidence of AC. MAC clinical and molecular features are distinct from AC and likely account for outcome differences. Overall, left-sided MAC was associated with the shortest OS. Molecular profiling may improve treatment guidelines for MAC.

## Introduction

Colon cancer represents the third most common malignancy among both men and women and is the second highest cause of cancer mortality in men and women, when combined, in the United States^1,2^. Early detection and improved treatments have resulted in not only improved overall survival in patients with colon cancer but also a decreased incidence in the United States^2^. Adenocarcinoma (AC) of the colon is the predominant histology, followed by neuroendocrine tumors and other rarer histologic entities^3^. In particular, medullary adenocarcinoma (MAC) is a very rare glandular tumor subtype of colon cancer^4,5^.

MAC was first described in the 1990’s^6^, but has historically been difficult to distinguish from poorly differentiated AC^5^. However, through identification of molecular markers specific to MAC^5,7,8^, MAC has been better defined and characterized leading to increased recognition and diagnosis in the last two decades. In published reports, the incidence rate of MAC is noted to be less than 1% of all primary colon cancers, with a preponderance in older women^9,10^. MAC is frequently characterized by features of aggressive tumor biology, such as lymphovascular invasion (LVI) and perineural invasion (PNI), as well as larger tumor size on presentation, yet with a decreased likelihood of progression to regional nodal and distant sites when compared with poorly differentiated AC^10–12^. At least part of the biological behavior of MAC may be explained by the finding that approximately 90% of MAC is associated with microsatellite instability (MSI)^6,10,13^.

Over the last two decades during which MAC has been recognized as a distinct subtype of colon cancer, there have been dramatic changes in our understanding and classification of colon cancer into integrated clinical and molecular colon cancer subtypes^14,15^. We analyzed data obtained through the Surveillance, Epidemiology, and End Results (SEER) program and the National Cancer Database (NCDB) to compare AC and MAC to better understand the incidence trends, clinicopathologic and treatment characteristics, and oncologic outcomes of MAC in the context of contemporary colon cancer management.

## Materials and methods

This study was exempt from institutional research board approval given the use and analysis of de-identified national datasets. Our study consisted of three dataset analyses: (1) age-adjusted incidence trends over time using SEER data, (2) patient characteristics of MAC versus AC using unmatched NCDB data, and (3) prognostic characteristics of MAC versus AC using NCDB data matched 1:1 on age, sex, race/ethnicity, number of Charlson comorbidities, laterality, and disease stage.

Trends in the annual age-adjusted incidence rates of MAC and AC were calculated using SEER*Stat. Included in our analyses were patients diagnosed with MAC or AC from 2002–2017. We chose to use SEER data and the SEER*Stat application because the dataset contains a module specifically designed for age-adjusted incidence rate calculations. For the cohort analyses examining clinicopathologic characteristics and cancer outcomes, we chose the NCDB dataset because, unlike SEER, Collaborative Staging site-specific factor data on *KRAS* and MSI are well-captured.

The SEER 21 dataset contains cancer incidence and survival information from 21 SEER sites and represents 36.7% of the United States population (https://seer.cancer.gov/registries/data.html#a7). From 2002 to 2017, in these 21 sites, 1,236 patients were diagnosed with MAC and 471,927 were diagnosed with AC. All patients with a malignant diagnosis of MAC or AC were included in the incidence rate analysis.

NCDB data were used to compare demographic, clinicopathologic, and outcome characteristics across MAC and AC. The NCDB is a joint project of the Commission on Cancer of the American College of Surgeons and the American Cancer Society. The patient population captured within the dataset represents 70% of those with new cancer diagnoses treated at the approximately 1,500 Commission on Cancer-designated Clinical Cancer Programs across the United States. The 2019 release of NCDB colon cancer Participant User File (PUF) was queried for all patients diagnosed with MAC or AC from 2010–2016.

**Figure 1:**
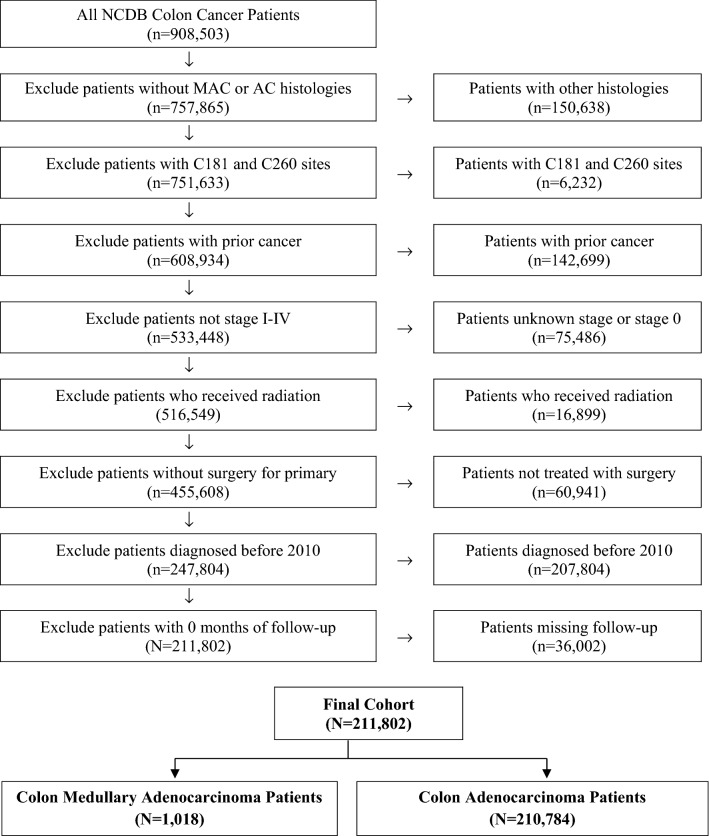
Stepwise patient selection criteria.

Given the de-identified nature of the data, this study was exempted from institutional review board approval. Figure 1 outlines the stepwise selection criteria for the NCDB patients. MAC patients were identified based on International Classification of Diseases for Oncology, 3^rd^ Edition (ICD-O-3) morphology codes 8510 and 8513 and cases of AC were identified using ICD-O-3 morphological codes 8140–8144, 8210–8213, 8220, and 8260–8263. Patient demographic, clinicopathologic, and treatment data were analyzed. Laterality of the primary tumor was delineated by ICD-O-3 topographical codes. Laterality was defined as “right” if the cancer was located from the cecum to the transverse colon (codes C180, C182-184) and defined as “left” if the cancer was located from the splenic flexure up to the rectosigmoid (codes C185-187). Overlapping or unspecified sites were indicated by codes C188 and C189, respectively. Patients with other histologic diagnoses were excluded. Our analyses were restricted to patients diagnosed from 2010 to coincide with the NCDB addition of Collaborative Staging site-specific factor data pertaining to microsatellite stability (MSS) or instability (MSI), and *KRAS* gene mutation status.

### Statistical analysis

Statistical analyses were performed using SEER*Stat 8.3.6^16^ and SAS version 9.4 TS Level 1M2 (SAS Institute, Cary, NC). Incidence rates were calculated using the number of cases over the number of individuals at risk in each of 19 age groups within the relevant SEER catchment area during a specific year. These rates were age adjusted using the 2000 US standard population (19 age groups; census P25-1130) and are expressed as per 100,000 individuals at risk^17^. Incidence rate changes over time are expressed as annual percent changes (APCs), which represent the log-transformed slopes across time.

To assess patient demographics and clinicopathologic characteristics, initially unmatched NCDB data were used. Due to the heterogeneity of the clinicopathologic features between MAC and AC, additional analyses of outcomes data were compared using a 1:1 matched analysis. The factors used to match the MAC patients with the AC patients included age (± 5 years), sex, race/ethnicity, number of comorbid conditions, laterality, and disease stage. All matching was performed using the GREEDY algorithm^18^.

Data were summarized using counts and percentages for categorical variables and median values with interquartile ranges (IQRs) for continuous data. Categorical data were compared using Pearson’s Χ^2^ analysis and continuous data were compared using the Wilcoxon rank-sum test. Univariate and multivariate Cox proportional hazards regression models were used to identify predictors of overall survival (OS). Multivariate OS results are depicted using forest plots. A two-sided *p* value of < 0.05 is considered significant.

## Results

### Annual incidence of MAC increased over time

**Figure 2:**
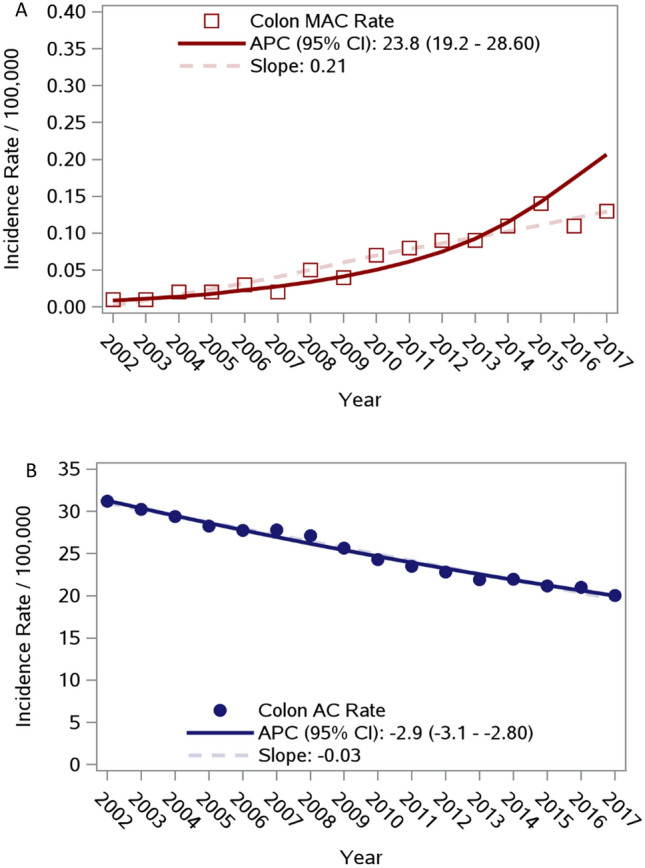
Colon medullary adenocarcinoma and adenocarcinoma incidence rates by histology. (**A**) Colon Medullary Adenocarcinoma. (**B**) Colon Adenocarcinoma.

From 2002–2017, the annual percent change (APC) in age-adjusted incidence rates of MAC increased by 23.8% (95% CI: 19.2–28.6) (Fig. 2A). In contrast, the APC in age-adjusted incidence rates of AC declined during the same time period (APC: − 2.9, 95% CI − 3.1 to − 2.8) (Fig. 2B).

### Demographic, clinicopathologic, and treatment characteristics of MAC differ from AC

#### Unmatched cohort

A cohort of 1,018 patients diagnosed with MAC and 210,784 diagnosed with AC were identified for analysis. Patient, clinicopathologic, and treatment characteristics are presented in Table 1. Compared to AC patients, those with MAC were older, predominantly female, and white (all *p* < 0.0001). The reported diagnosis of MAC increased over time, with 50% of patients identified in the last four years of data collection (data not shown). MAC was located more often in the right colon; more frequently classified as high tumor grade and with LVI; and more likely to be Stage II disease (all *p* < 0.0001). Although such high-risk features were more commonly present in MAC, patients with MAC had lower rates of regional lymph node involvement (*p* = 0.037). Of patients with specified microsatellite status, over 81% of MAC patients had MSI versus 24% of AC patients (*p* < 0.0001). Among patients with known *KRAS* mutation status, MAC was associated with lower rates of *KRAS*-mutant disease (*p* < 0.0001). Overall, chemotherapy was administered to AC patients with greater frequency than to patients with MAC (*p* < 0.0001).

**Table 1:** Baseline demographic and clinicopathologic characteristics of participants by histology status.

		Adenocarcinoma N = 210,784 N (%)	Medullary Adenocarcinoma N = 1018 N (%)	Odds ratio (95% CI)	*p*-value
**Patient characteristics**					
Age at diagnosis	Median (IQR^+^)	68 (57–77)	75 (66–83)	1.03 (1.03–1.04)	< 0.0001
Age at diagnosis	18–49	21,843 (10)	71 (7)	-Ref-	–
	50–64	65,951 (31)	159 (16)	0.74 (0.56–0.98)	0.0367
	65–79	80,311 (38)	417 (41)	1.60 (1.24–2.06)	0.0003
	≥ 80	42,679 (20)	371 (36)	2.67 (2.07–3.45)	< 0.0001
Sex	Male	103,334 (49)	255 (25)	-Ref-	–
	Female	107,450 (51)	763 (75)	2.88 (2.50–3.32)	< 0.0001
Race/ethnicity	Non-hispanic white	161,360 (77)	881 (87)	-Ref-	–
	Black	27,493 (13)	55 (5)	0.37 (0.28–0.48)	< 0.0001
	Hispanic white	10,720 (5)	36 (4)	0.62 (0.44–0.86)	0.0043
	Other	9892 (5)	34 (3)	0.63 (0.45–0.89)	0.0082
	Unknown	1319 (1)	12 (1)	1.67 (0.94–2.95)	0.0800
Charlson comorbidities	None	144,120 (68)	671 (66)	-Ref-	–
	1	46,967 (22)	239 (23)	1.09 (0.94–1.27)	0.2391
	> = 2	13,501 (6)	67 (7)	1.07 (0.83–1.37)	0.6193
	Unknown	6196 (3)	41 (4)	1.42 (1.04–1.95)	0.0294
**Clinicopathologic characteristics**					
Laterality	Right	124,830 (59)	904 (89)	-Ref-	–
	Left	79,762 (38)	82 (8)	0.14 (0.11–0.18)	< 0.0001
	Overlapping/NOS	6192 (3)	32 (3)	0.71 (0.50–1.02)	0.0614
Lymph vascular invasion	Not present	132,271 (63)	521 (51)	-Ref-	–
	Present	59,699 (28)	441 (43)	1.88 (1.65–2.13)	< 0.0001
	Not applicable	14 (0)	0 (0)	UND**	0.9576
	Unknown	18,800 (9)	56 (6)	0.76 (0.57–1.00)	0.0473
Perineural invasion	No perineural invasion	166,671 (79)	812 (80)	-Ref-	–
	Perineural invasion	25,855 (12)	148 (15)	1.17 (0.99–1.40)	0.0720
	Unknown	18,258 (9)	58 (6)	0.65 (0.50–0.85)	0.0017
Grade	I or II	165,083 (78)	40 (4)	0.01 (0.01–0.02)	< 0.0001
	III	32,560 (15)	673 (66)	-Ref-	–
	IV	5621 (3)	237 (23)	2.04 (1.75–2.37)	< 0.0001
	Unknown	7520 (4)	68 (7)	0.44 (0.34–0.56)	< 0.0001
Node status	N −	121,611 (58)	621 (61)	-Ref-	–
	N +	86,982 (41)	388 (38)	0.87 (0.77–0.99)	0.0371
	Unknown	2191 (1)	9 (1)	0.80 (0.42–1.56)	0.5178
Stage (AJCC)	Stage I	49,334 (23)	129 (13)	-Ref-	–
	Stage II	64,910 (31)	458 (45)	2.70 (2.22–3.28)	< 0.0001
	Stage III	68,710 (33)	360 (35)	2.00 (1.64–2.45)	< 0.0001
	Stage IV	27,830 (13)	71 (7)	0.98 (0.73–1.30)	0.8679
Examined nodes	0	1372 (1)	6 (1)	-Ref-	–
	1–11	22,928 (11)	55 (5)	0.55 (0.24–1.28)	0.1635
	> = 12	185,521 (88)	952 (94)	1.17 (0.52–2.62)	0.6968
	Unknown	963 (0)	5 (0)	1.19 (0.36–3.90)	0.7773
**Treatment characteristics**					
Chemotherapy	No chemo given	123,766 (59)	689 (68)	-Ref-	–
	Chemo received	79,707 (38)	294 (29)	0.66 (0.58–0.76)	< 0.0001
	Unknown	7311 (3)	35 (3)	0.86 (0.61–1.21)	0.3850
**Molecular studies**					
Microsatellite status	Microsatellite stability	41,869 (20)	82 (8)	-Ref-	–
	Microsatellite instability	13,302 (6)	364 (36)	13.96 (10.98–17.75)	< 0.0001
	Unknown	155,613 (74)	572 (56)	1.88 (1.49 –2.36)	< 0.0001
*KRAS* status	Normal	16,109 (8)	124 (12)	-Ref-	–
	Abnormal	10,692 (5)	25 (2)	0.30 (0.20–0.47)	< 0.0001
	Unknown	183,983 (87)	869 (85)	0.61 (0.51–0.74)	< 0.0001

#### Matched cohort

Included in the matched results were 1016 MAC patients matched 1:1 with AC patients on age, sex, race, number of comorbidities, laterality, and disease stage. Patient demographic, clinicopathologic, and treatment characteristics are presented in Table S1. Univariate analyses of matched variables showed good matching of the groups (*p* > 0.95 for all matched variables). Compared to AC, MAC lesions were significantly more likely to be high-grade with LVI, MSI, and wild-type *KRAS* (all *p* < 0.0001).

**Figure 3:**
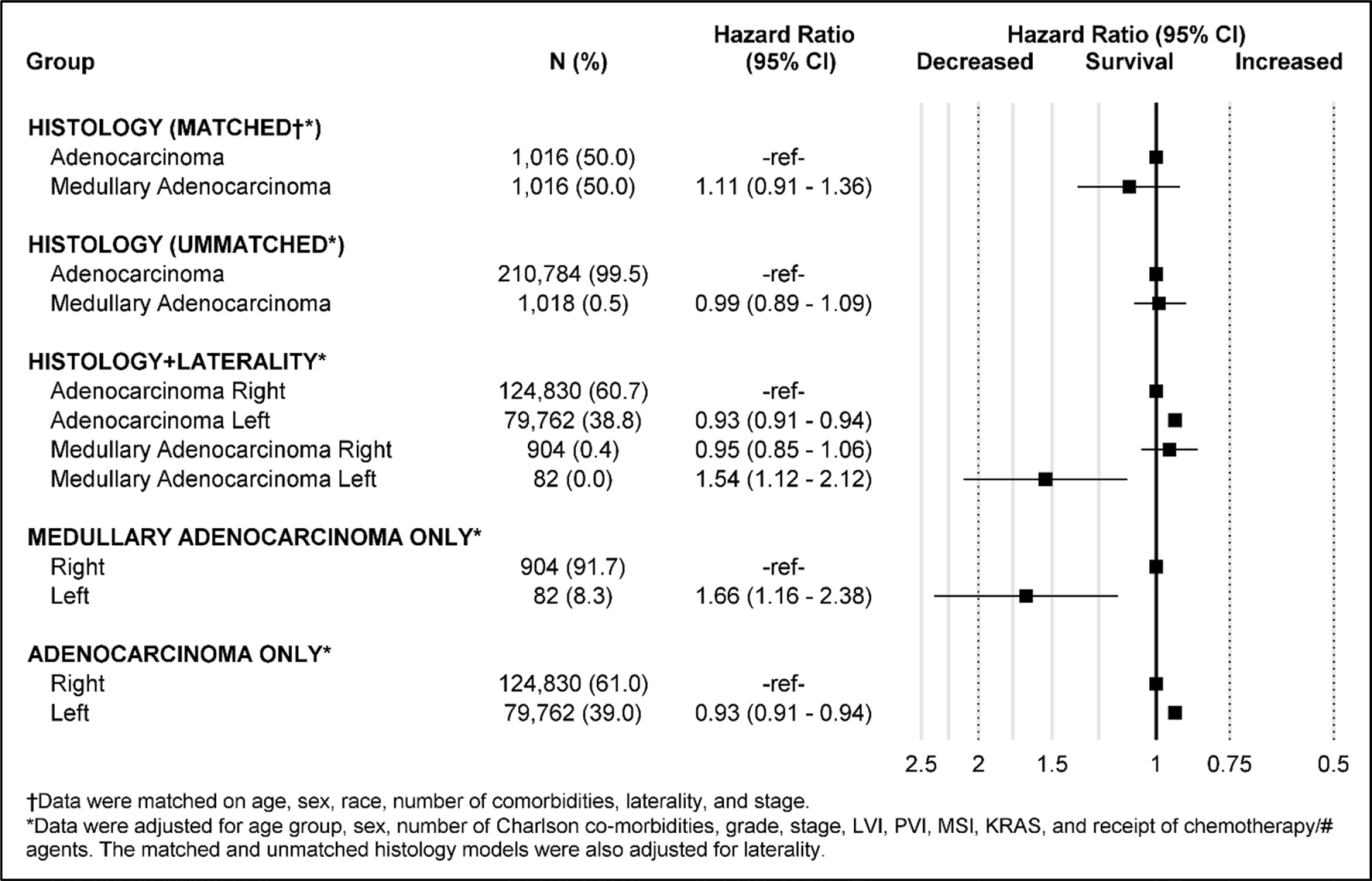
Overall survival by histology and laterality of left and right-sided colon medullary adenocarcinoma and colon adenocarcinoma.

Multivariate Cox proportional hazards regression was used to look at independent risk factors for overall survival (OS). A forest plot depicting multivariate results is shown in Fig. 3. After adjusting for age group, sex, number of Charlson co-morbidities, grade, stage, laterality, LVI, PVI, MSI, *KRAS*, and receipt of chemotherapy, MAC was not independently associated with overall survival (OS) (HR 1.11, 95% CI 0.91–1.35).

### Overall survival depends on laterality of subtypes

After stratifying the histologic groups by laterality, multivariate results on the unmatched group showed left-sided MAC tumors were associated with significantly shorter survival when compared to right-sided MAC (HR 1.66, 95% CI 1.16 − 2.38) and right-sided AC (HR 1.54, 95% CI 1.12–2.12) (Fig. 3). In contrast, left-sided AC was associated with significantly longer survival when compared to right -sided AC tumor (HR 0.93, 95% CI 0.91–0.94).

## Discussion

Colon MAC is a rare subtype of colon cancer. Previous publications have estimated that the disease represents less than 1% of all colon cancers^6,10^. In our analysis of the SEER dataset, we confirmed the rarity of this disease. However, over the last two decades there has been an increase in the incidence of reported cases of MAC with an APC of 23.8%. Whether this rise in incidence is as a result of increased recognition and reporting of the histologic entity or due to actual increased number of cases in the population remains uncertain. That the majority of the cases captured have been in the last 4 years of our analysis suggests that better recognition of the entity, perhaps through immunohistochemical tests for mismatch repair (MMR) proteins and confirmation of MSI, may account for the incidence rise. Regardless, our finding that the incidence of MAC is rising argues that a better understanding of the clinical characteristics and oncologic outcomes of MAC, in comparison to AC, is needed to better tailor treatment to this distinct colon cancer histologic subtype.

Clinical characteristics of MAC have been previously described in small single institution studies and in large public dataset analyses^4,9,11^. There are distinct clinical features associated with MAC when compared to AC. MAC is more frequently characterized by local aggressiveness, including more advanced T-stage, higher histopathologic grade, and presence of LVI and PNI, although with less likelihood of nodal or distant involvement. Furthermore, MAC is predominantly found in the right colon (89%), with far higher frequency of MSI (81%) than would be expected for right-sided colon ACs.

The molecular characteristic that distinguishes MAC from AC is the predisposition to MSI^5,6^. As shown in our data and in support of the other studies, more than 80% of MAC patients with reported microsatellite status were positive for MSI. Microsatellite instability, first described in 1993, represents deficiency of the mismatch repair system (dMMR phenotype)^19^. Hereditary nonpolyposis colorectal cancer, a component of Lynch syndrome, is a consequence of germline mutations in one of the MMR genes, *MLH1*, *MSH2*, *MSH6*, and *PMS2*^20^. In contrast, sporadic cases of colon cancer with MSI develop from senescent hypermethylation of the *MLH1* gene promoter^21^. The clinical relevance of these differing etiologies has been explored, suggesting distinct tumor biology^22,23^. Although the NCDB does not have discrete information regarding sporadic or inherited etiology of MSI status, which would have enriched our analysis, the vast majority of MSI patients in our cohort were likely sporadic, especially given the older age at diagnosis, the sex distribution favoring female patients, and the overwhelmingly right-sided disease^24^.

MSI has been associated with improved OS in colon AC^19^. However, it is unclear whether colon MAC, given the high preponderance of MSI that is the hallmark of the subtype, likewise experiences improved OS compared to MSS MAC or AC. In the early description by Lanza et al., MAC was identified to be a distinct subtype with improved OS^6^. Subsequent studies, including a previous publication using the NCDB dataset, likewise show better OS for MAC^9^. However, in a recent publication from Gomez-Alvarez, OS was worse for MAC when compared with MSI high AC in a small, single institutional series^11^. To better understand the survival outcomes of MAC when compared to AC, we performed survival analyses. We find that overall survival was similar between MAC and AC in the NCDB dataset. To limit potential bias, we further analyzed the data by conducting a survival analysis of a 1:1 matched cohort and confirmed that overall survival was similar among MAC compared to AC (HR 1.11, 95% CI 0.91–1.35).

Sidedness or laterality in colon cancer is increasingly recognized as an important prognostic factor. A meta-analysis of colorectal cancer studies stratified by sidedness reported a 19% reduction in mortality for left-sided malignancies compared to right-sided cancers^25^. Embryologically, the colon develops from both the midgut and hindgut, which in conjunction with the evolution of the host’s microbiome, may lead to unique genetic phenotypes between right- and left-sided tumors^14,26^. Treatment strategies for colon cancer have evolved to account for differential genetic expression profiles by tumor location. One example of this is anti-epidermal growth factor receptor therapies for wild-type *KRAS* colon cancer, which have demonstrated a preferential survival benefit for left-sided cancers^27,28^. To evaluate the effect of sidedness on overall survival in colon AC and MAC patients, we grouped the unmatched dataset into right-sided AC, left-sided AC, right-sided MAC, and left-sided MAC. Compared to right-sided AC, improved survival was seen among left-sided AC (HR 0.93, 95% CI 0.91–0.94). In contrast, compared to right-sided AC, decreased survival was seen in left-sided MAC (HR 1.54, 95% CI 1.12–2.12). Similarly, when looking at MAC patients only, those with left-sided cancers had significantly worse survival than those with right-sided cancers (HR 1.66, 95% CI 1.16-2.38).

At present, resection remains upfront treatment for colon cancer regardless of subtype. However, nuances in adjuvant treatment strategies for stage II and III disease and systemic treatment regimens for stage IV disease are increasingly based on molecular markers such as MSI and *KRAS* status. Although we demonstrate an improvement in the survival HR in MAC patients who are treated with chemotherapy, the lack of granularity in public datasets limit the ability to characterize the agents and the regimens associated with that benefit.

The well-described limitations of databases such as the NCDB include the lack of disease recurrence data and cause of death. However, the use of a large, public dataset is necessary to aggregate adequate numbers of rare cancers such as MAC for analysis. Other limitations include data availability. Specific to our study, important tumor factors such as MSI, LVI, and *KRAS* status have only been collected since 2010, limiting the length of follow-up available for analysis. In addition, the low numbers of known *KRAS* and *BRAF* mutation information documented in the database precluded a more detailed analysis of MSI, *KRAS*, and *BRAF* as prognostic factors for MAC versus AC. Specific systemic treatment-related analysis remains limited by the demonstrated low sensitivity of records of such treatments in the NCDB^29^. Very few patients were recorded as having received immunotherapy, and a substantial minority of patients with stage III–IV disease were not noted to have received chemotherapy; suggesting both data points were likely under-reported. In addition, the NCDB lacks information on administration of targeted therapy in addition to cytotoxic chemotherapy.

## Conclusions

Colon medullary adenocarcinoma is increasingly recognized and diagnosed. Medullary histology closely resembles microsatellite-unstable poorly differentiated colon adenocarcinoma with pathologic features of local aggressiveness such as larger tumor size and LVI. However, left-sided MAC demonstrates worse overall survival compared to right-sided MAC and bilateral AC. Efforts made to distinguish MAC from AC at the molecular level may improve on the ability to define each entity, when further stratified by sidedness, to determine the prognostic impact of *KRAS* and *BRAF* mutations in the setting of MSI, and to identify other prognostic and predictive molecular markers to develop more specific treatment guidelines for this rare cancer.

## Supplementary Information

Supplementary Information.
