# Supplementary material for: Sidedness determines clinical characteristics and survival outcomes in medullary adenocarcinoma of the colon
Source: Sci Rep. 2021 Oct 14;11:20481. doi: 10.1038/s41598-021-99848-y (PMC8516966; doi:10.1038/s41598-021-99848-y)
Supplement: Supplementary file 1 — Supplementary Information. [file 41598_2021_99848_MOESM1_ESM.docx]

Table S1. Baseline demographic and clinicopathologic characteristics of 1:1 matched* participants by histology status.

|  | | **Adenocarcinoma N=1,016 N (%)** | **Medullary Adenocarcinoma N=1,016 N (%)** | **Odds Ratio (95% CI)** | **p-value** |  |
| --- | --- | --- | --- | --- | --- | --- |
| **Patient Characteristics** | | | | | | |
| Age at diagnosis | Median (IQR^+^) | 75 (66-83) | 75 (66-83) | 1.00 (0.99 - 1.01) | 0.9933 |  |
| Age at diagnosis | 18-49 | 71 (7) | 71 (7) | -ref- | - |  |
|  | 50-64 | 159 (16) | 159 (16) | 1.00 (0.67 - 1.49) | 1.0000 |  |
|  | 65-79 | 415 (41) | 415 (41) | 1.00 (0.70 - 1.43) | 1.0000 |  |
|  | ≥80 | 371 (37) | 371 (37) | 1.00 (0.70 - 1.43) | 1.0000 |  |
| Sex | Male | 255 (25) | 255 (25) | -ref- | - |  |
|  | Female | 761 (75) | 761 (75) | 1.00 (0.82 - 1.22) | 1.0000 |  |
| Race/ethnicity | Non-Hispanic White | 881 (87) | 881 (87) | -ref- | - |  |
|  | Black | 55 (5) | 55 (5) | 1.00 (0.68 - 1.47) | 1.0000 |  |
|  | Hispanic White | 36 (4) | 36 (4) | 1.00 (0.62 - 1.60) | 1.0000 |  |
|  | Other | 32 (3) | 32 (3) | 1.00 (0.61 - 1.65) | 1.0000 |  |
|  | Unknown | 12 (1) | 12 (1) | 1.00 (0.45 - 2.24) | 1.0000 |  |
| Charlson comorbidities | None | 671 (66) | 671 (66) | -ref- | - |  |
|  | 1 | 239 (24) | 239 (24) | 1.00 (0.81 - 1.23) | 1.0000 |  |
|  | >=2 | 65 (6) | 65 (6) | 1.00 (0.70 - 1.43) | 1.0000 |  |
|  | Unknown | 41 (4) | 41 (4) | 1.00 (0.64 - 1.56) | 1.0000 |  |
| **Clinicopathologic Characteristics** | | | | | | |
| Laterality | Right | 903 (89) | 903 (89) | -ref- | - |  |
|  | Left | 81 (8) | 81 (8) | 1.00 (0.73 - 1.38) | 1.0000 |  |
|  | Overlapping/NOS | 32 (3) | 32 (3) | 1.00 (0.61 - 1.65) | 1.0000 |  |
| Lymph vascular invasion | Not present | 643 (63) | 519 (51) | -ref- | - |  |
|  | Present | 300 (30) | 441 (43) | 1.82 (1.51 - 2.20) | <.0001 |  |
|  | Unknown | 73 (7) | 56 (6) | 0.95 (0.66 - 1.37) | 0.7858 |  |
| Perineural invasion | No perineural invasion | 812 (80) | 811 (80) | -ref- | - |  |
|  | Perineural invasion | 128 (13) | 147 (14) | 1.15 (0.89 - 1.49) | 0.2853 |  |
|  | Unknown | 76 (7) | 58 (6) | 0.76 (0.54 - 1.09) | 0.1378 |  |
| Grade | I or II | 728 (72) | 40 (4) | 0.02 (0.01 - 0.03) | <.0001 |  |
|  | III | 215 (21) | 671 (66) | -ref- | - |  |
|  | IV | 47 (5) | 237 (23) | 1.62 (1.14 - 2.29) | 0.0070 |  |
|  | Unknown | 26 (3) | 68 (7) | 0.84 (0.52 - 1.35) | 0.4681 |  |
| Node status | N- | 603 (59) | 620 (61) | -ref- | - |  |
|  | N+ | 407 (40) | 387 (38) | 0.92 (0.77 - 1.11) | 0.3911 |  |
|  | Unknown | 6 (1) | 9 (1) | 1.46 (0.52 - 4.12) | 0.4762 |  |
| Stage (AJCC) | Stage I | 128 (13) | 128 (13) | -ref- | - |  |
|  | Stage II | 458 (45) | 458 (45) | 1.00 (0.76 - 1.32) | 1.0000 |  |
|  | Stage III | 360 (35) | 360 (35) | 1.00 (0.75 - 1.33) | 1.0000 |  |
|  | Stage IV | 70 (7) | 70 (7) | 1.00 (0.66 - 1.51) | 1.0000 |  |
| Examined nodes | 0 | 3 (0) | 6 (1) | -ref- | - |  |
|  | 1-11 | 74 (7) | 55 (5) | 0.37 (0.09 - 1.55) | 0.1747 |  |
|  | >=12 | 937 (92) | 950 (94) | 0.51 (0.13 - 2.03) | 0.3378 |  |
|  | Unknown | 2 (0) | 5 (0) | 1.25 (0.15 - 10.69) | 0.8390 |  |
| **Treatment Characteristics** | | | | | | |
| Chemotherapy | No chemo given | 672 (66) | 688 (68) | -ref- | - |  |
|  | Chemo received | 297 (29) | 293 (29) | 0.96 (0.79 - 1.17) | 0.7068 |  |
|  | Unknown | 47 (5) | 35 (3) | 0.73 (0.46 - 1.14) | 0.1659 |  |
| **Molecular Studies** | | | | | | |
| Microsatellite status | Microsatellite stability | 171 (17) | 82 (8) | -ref- | - |  |
|  | Microsatellite instability | 89 (9) | 363 (36) | 8.51 (5.99 - 12.08) | <.0001 |  |
|  | Unknown | 756 (74) | 571 (56) | 1.58 (1.18 - 2.09) | 0.0018 |  |
| KRAS | Normal | 67 (7) | 124 (12) | -ref- | - |  |
|  | Abnormal | 43 (4) | 25 (2) | 0.31 (0.18 - 0.56) | <.0001 |  |
|  | Unknown | 906 (89) | 867 (85) | 0.52 (0.38 - 0.71) | <.0001 |  |
| *Data were matched on age, sex, race, number of comorbidities, laterality, and stage. Matching on KRAS was not performed due to the high percentage of missing values. | | | | | | |
